# A systems biology framework for modeling metabolic enzyme inhibition of *Mycobacterium tuberculosis*

**DOI:** 10.1186/1752-0509-3-92

**Published:** 2009-09-15

**Authors:** Xin Fang, Anders Wallqvist, Jaques Reifman

**Affiliations:** 1 Biotechnology HPC Software Applications Institute, Telemedicine and Advanced Technology Research Center, U.S. Army Medical Research and Materiel Command, Ft. Detrick, MD 21702, USA

## Abstract

**Background:**

Because metabolism is fundamental in sustaining microbial life, drugs that target pathogen-specific metabolic enzymes and pathways can be very effective. In particular, the metabolic challenges faced by intracellular pathogens, such as *Mycobacterium tuberculosis*, residing in the infected host provide novel opportunities for therapeutic intervention.

**Results:**

We developed a mathematical framework to simulate the effects on the growth of a pathogen when enzymes in its metabolic pathways are inhibited. Combining detailed models of enzyme kinetics, a complete metabolic network description as modeled by flux balance analysis, and a dynamic cell population growth model, we quantitatively modeled and predicted the dose-response of the 3-nitropropionate inhibitor on the growth of *M. tuberculosis *in a medium whose carbon source was restricted to fatty acids, and that of the 5'-*O*-(*N*-salicylsulfamoyl) adenosine inhibitor in a medium with low-iron concentration.

**Conclusion:**

The predicted results quantitatively reproduced the experimentally measured dose-response curves, ranging over three orders of magnitude in inhibitor concentration. Thus, by allowing for detailed specifications of the underlying enzymatic kinetics, metabolic reactions/constraints, and growth media, our model captured the essential chemical and biological factors that determine the effects of drug inhibition on *in vitro *growth of *M. tuberculosis *cells.

## Background

System-level networks of biological processes and functions allow us to draw inferences about the phenotype of an organism that cannot be made by considering each of its individual components [1-4]. In particular, metabolic networks are made up of hundreds to thousands of distinct but interconnected chemical reactions, each processing particular metabolites in different locations of the cell that, taken together, ultimately allow the cell to function and grow [5]. The metabolic network of an organism is assembled, through automated and manual procedures [6,7], based on known chemical reactions collected from genome annotation databases, such as the Kyoto Encyclopedia of Genes and Genomes [8]. Currently, assemblies of metabolic networks are available for several bacterial species [9-13], yeast [14], and humans [15].

Several quantitative approaches have been developed to study different aspects of metabolic networks [16,17]. Kinetic models comprised of explicit sets of reactants and reactions can be constructed and solved via ordinary differential equations (ODEs), provided the rate constants for each reaction are known. Kinetic models are typically restricted to a selected set of reactions used to study, for example, metabolism in human red blood cells [18], core components of the anaerobic metabolism in *Escherichia coli *[19], and the relation between single nucleotide polymorphisms and anemia [20]. Kinetics of inhibiting enzymes can be incorporated into such models [21]. However, due to the limited number of reactions modeled, only parts of the metabolic network can be taken into account and the whole organism's response (i.e., its phenotype) to the inhibitor cannot be modeled. In more comprehensive studies of the entire metabolic network of organisms for which kinetic information of all reactions is not available, flux balance analysis (FBA) can be used to obtain the optimal steady-state reaction flux distribution and the organism's growth rate under constraints imposed on the directions of the reactions, stoichiometry, and maximal transport fluxes [22]. FBA can predict experimentally determined cellular growth rates in different media [10-12,23-25] and identify genes, which, when removed, prevent cellular growth [10,11,14,26-29]. Going beyond steady-state conditions of the traditional FBA, cell growth dynamics can be taken into account in a dynamic flux balance model [25,30]. Such dynamic flux balance models provide a link between temporal changes of nutrient concentrations in a given medium and cell growth, as nutrients are consumed by the cell. Because these conditions are typically encountered in experimental studies of bacterial growth, dynamic flux balance models provide an important mechanism for understanding and representing experimental observations.

Enzyme inhibition kinetics, FBA of metabolic networks, and cell growth dynamics have each been studied separately before. Moreover, enzyme inhibition kinetics has been incorporated into a portion of a metabolic network [21], and FBA of a metabolic network has been combined with cell growth dynamics [30]. However, integration of all three components has not been attempted before. Here, we present a framework that links together all these three components - enzyme inhibition kinetics, FBA of a metabolic network, and cell growth dynamics - to model the growth inhibition of *Mycobacterium tuberculosis*, the causative agent of tuberculosis (TB).

TB is a major infectious disease in the world with over 9.2 million new cases and 1.7 million deaths in 2006, and it is estimated that one-third of the human population is infected with the disease [31]. Mycobacteria are aerobic organisms classified as acid-fast Gram-positive bacteria due to their lack of an outer cell membrane. They are a relatively slowly dividing organism compared with other bacteria. Most treatments for tuberculosis directly interfere with mycobacteria-specific physiology [32]. *M. tuberculosis *is a prototrophic and metabolically flexible organism capable of surviving in a variety of environments. Bacteria that reach the lung alveoli are internalized by resident macrophages, where they are able to replicate in modified vacuoles [33-35]. At the onset of adaptive immunity, activated macrophages keep the infection under control, but the bacteria are not eliminated, and a state of chronic persistence is established [36]. Survival under such conditions requires metabolically active bacteria capable of producing counter-immune effectors [34,37,38].

Worldwide efforts to eliminate TB are confronting many obstacles, including drug-resistant pathogens, compliance with complicated drug regimens, and compromised immune systems associated with human immunodeficiency syndrome or acquired immunodeficiency syndrome [39]. Partly to address these issues, renewed efforts have begun in developing drugs that target the intracellular metabolism of *M. tuberculosis*, for example, by analyzing metabolic pathways to identify potential drug targets that selectively affect *M. tuberculosis *[40]. Importantly, using the sequenced genome of *M. tuberculosis *[41] together with literature data on known metabolic reactions, extensive metabolic network reconstructions have been carried out for this organism [42,43]. Analyses of these networks based on FBA reveal that they contain sufficient information to predict growth rates and identify genes that are essential for the growth of *M. tuberculosis *in select media [42,43].

Novel drug design approaches against *M. tuberculosis *metabolism exploit the unique and harsh conditions that the pathogen is exposed to in the host environment. After entering a host, pathogens are confronted with a nutrient-poor environment and are often restricted to utilizing fatty acids as their main carbon source [32,38]. This is accomplished by activation of the glyoxylate shunt pathway and the methylcitrate cycle [44,45]. Consequently, the ability to inhibit key reactions of these two pathways makes 3-nitropropionate (3-NP) an effective inhibitor for the *in vitro *growth of *M. tuberculosis *in fatty acid media as well as for its *in vivo *growth in mouse macrophage cells [46]. In addition to presenting a limited carbon source, the host environment is also deficient in iron, another nutritional requirement for the invading pathogen. Free iron is strictly controlled in the host environment via host iron-binding proteins, such as human transferrin, as a way to defend against bacterial infections [47]. Thus, many pathogens synthesize siderophores, chemicals with very high affinity for iron, to wrestle iron away from the host [48]. For *M. tuberculosis*, mycobactin is the necessary siderophore required for growth in macrophages and media containing low concentrations of iron [49]. Therefore, the biosynthesis of mycobactin [50] and the regulation of this iron uptake mechanism [51,52] have been extensively studied as a potential drug target. This led to the discovery that 5'-*O*-(*N*-salicylsulfamoyl) adenosine (sAMS) can function as an inhibitor to *M. tuberculosis *via its ability to inhibit mycobactin synthesis [53].

Here we developed a mathematical framework in which enzyme inhibition kinetics, metabolic network simulation, and cell growth dynamics are considered together to produce a system that is able to quantitatively model drug inhibition of cell growth. We separately simulated the effects of two metabolic inhibitors, 3-NP and sAMS, on the growth of *M. tuberculosis *cells, using an *in vitro *media model designed to mimic the limited nutritional environment in a host cell. The predicted dose-response curves quantitatively reproduced the observed experimental data, indicating that the developed modular framework was capable of capturing the effects of metabolic inhibitors on bacterial cell growth.

## Methods

The mathematical framework needed to map the amount of drug to the collective growth response of the *M. tuberculosis *bacterium required that we connect enzyme inhibition kinetics, metabolic network modeling, and bacterial population growth models. If these components could be modeled and verified by experimental data, we could create a computational system to quantitatively predict how metabolic inhibitors affect bacterial growth. Here, we present the framework that allowed us to generate and reproduce the dose-response curves of two metabolic inhibitors generated from two independent experimental studies.

**Figure 1:**
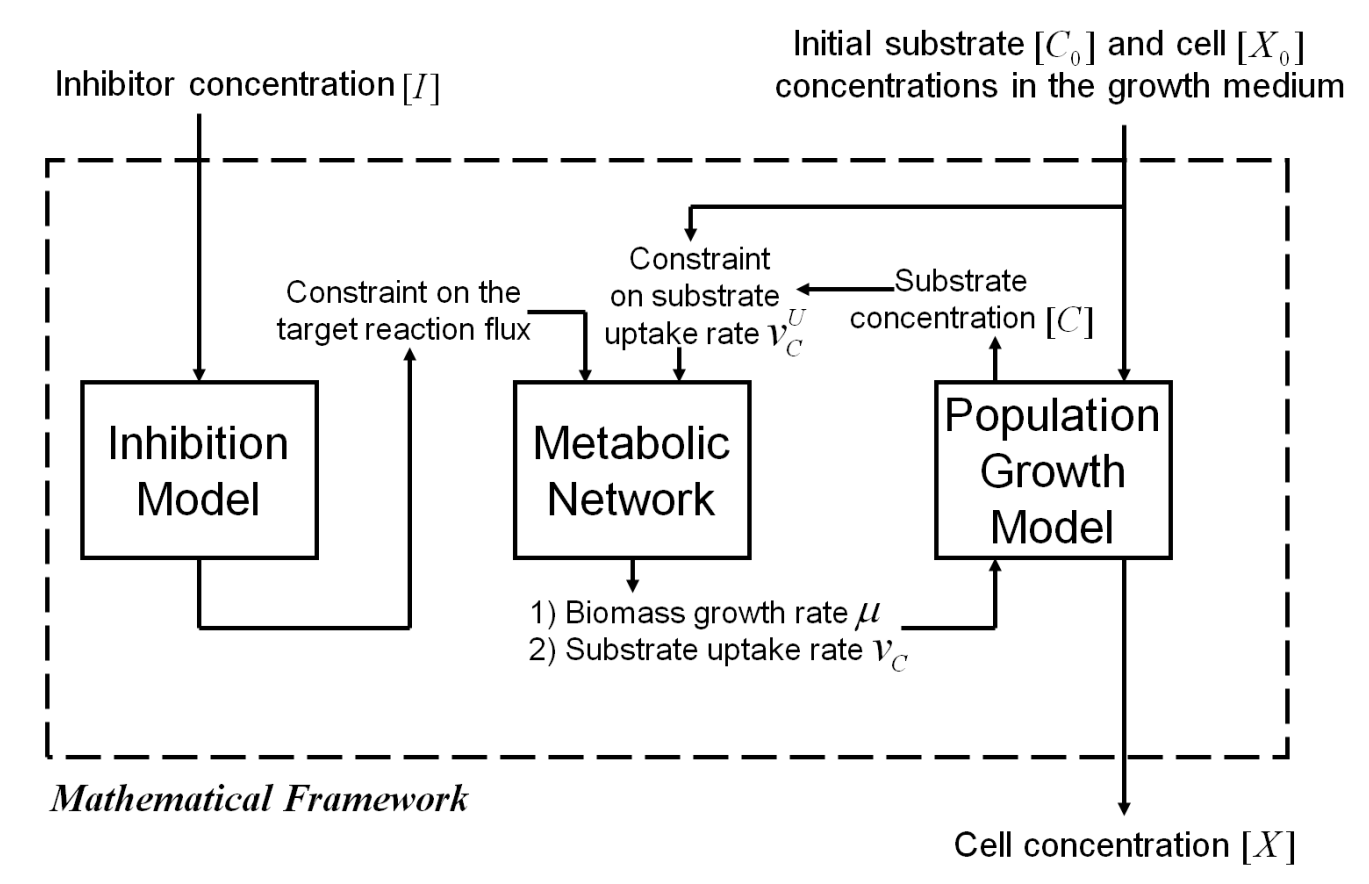
**A schematic view of the framework to simulate an inhibitor's effect on bacterial growth**. Given the inhibitor concentration [*I*], the Inhibition Model describes how the inhibitor affects the reaction flux of the reaction being inhibited (i.e., the target reaction). These effects are modeled via explicit constraints on the target reaction flux. Using these constraints and the constraints on substrate uptake rate , we analyzed the Metabolic Network to infer the biomass growth rate *μ *and substrate uptake rate *v*_*C*_. Using the Population Growth Model, we related biomass growth rate *μ *and substrate uptake rate *v*_*C *_to cell concentration [*X*]. We dynamically coupled the biomass growth rate and the diminished substrate concentration to develop a time-dependent model that dynamically infers cell concentration after the introduction of an inhibitor. Once these model components were specified, together with the initial substrate [C_0_] and cell [*X*_0_] concentrations in the growth medium, the calculations performed within this framework only required input in the form of a specific inhibitor concentration [*I*] to predict cellular growth.

The mathematical framework provides the connection between a) how a particular inhibitor affects the flux(es) of one or more metabolic reactions [Inhibition Model] (the affected reactions are referred to as target reactions), b) how the change in the metabolite flow or flux of the target reactions decreases the growth rate of the organism [Metabolic Network], and, finally, c) how the reduced growth rate results in an effective lower bacterial cell concentration [Population Growth Model]. Figure 1 schematically shows these three components and how they connect to and depend on each other. With the models specified and connected as outlined in Figure 1, the computational procedure only depends on the inhibitor concentration and the initial substrate and cell concentrations in the medium under which the organism was grown to calculate the subsequent bacterial cell concentration. The details specifying the internal workings of each model are given below.

### Inhibition model

The *inhibition model *is defined by the enzyme inhibition kinetics governing the reactants and products of a particular metabolic reaction (i.e., the target reaction) and relates the manner by which the inhibitor concentration [*I*] modifies or adds constraint to the flux of the target reaction. The mathematical form of the inhibition model depends on the particular enzyme kinetics associated with the specific inhibitor and the metabolic reaction affected by the inhibitor. Mathematically, the inhibition model relates an inhibitor concentration [*I*] to the resulting metabolite flux ratio in the presence and absence of the inhibitor. Inhibitors affecting more than one reaction can also be considered.

### Metabolic network

The *metabolic network *is used to self-consistently calculate the overall biomass growth rate *μ*, substrate uptake rates *v*_*C*_, and the fluxes of all metabolic reactions. It is coupled to the *inhibition model *and the *population growth model*. The *inhibition model *places constraints on the flux of the target reactions in the metabolic network that affects the total biomass growth rate, and the *population growth model *adds constraints to the network's substrate uptake rate from the medium. The effect of enzyme deletions (deletion mutants) on growth can be incorporated in the metabolic network model by removal of the particular reactions catalyzed by the specified enzymes [22,54].

The metabolic network developed by Jamshidi and Palsson [42] for *M. tuberculosis*, *iNJ*661, is able to quantitatively reproduce observed growth rates under a number of different conditions. We used this network, with some modifications, as the basis for our work (see Additional file 1: Section S1) and verified that the modifications did not affect the growth rate of *M. tuberculosis*, as originally reported [42].

The rate of growth of the biomass, the substrate uptake rates, and the reaction fluxes were obtained directly from the metabolic network by applying the FBA [26]. By using a linear programming method, FBA can maximize the biomass growth rate subject to steady-state mass balance of all the intracellular metabolites, and the stoichiometric constraints defined by the reactions. The maximization of biomass growth rate is based on the assumption that bacteria maximize their growth during the exponential stage and early stationary stage conditions. This assumption has been shown by previous studies to generate experiment-compatible results under such conditions [25,30]. Additional constraints that can be modeled in the FBA include specification of reversible and irreversible reactions, as well as limits placed on substrate uptake and target reaction rates. Here, FBA was performed with the COBRA Toolbox [55]. We also used the COBRA Toolbox to minimize the reaction fluxes while keeping the calculated maximal biomass growth rate. This procedure allowed us to obtain a unique set of minimum fluxes corresponding to the most parsimonious flow of metabolites through the network [56-58]. These fluxes were then used to constrain the target reaction rates.

### Population growth model

Given the biomass growth rate *μ *and the substrate uptake rates *v*_*C *_defined by the metabolic network, the *population growth model *provides a mechanism for calculating cell [*X*] and substrate [*C*] concentrations in the specified medium. This model considers changes in the substrate concentrations over time, which could be used to monitor how different carbon sources are preferentially used in the metabolic process [30].

Mathematically, the population growth model links the biomass growth rate *μ *to the actual cell concentration [*X*] of the bacteria as a function of time *t*. This process needs to consider the temporal depletion of the limiting substrate *C *in the medium as cells grow, which is dependent of the substrate uptake rate *v*_*C *_by the cells. Because the more cells grow the more they consume the limiting substrate, we represented this coupling through the following two ODEs:

(1)

(2)

where the brackets [.] indicate the concentration of "." and [*C*] represents the concentration of the limiting substrate *C *in the medium. The factor 24 simply converts the time *t *from hours to days, since the units of *μ *and *v*_*C *_are expressed, respectively, in h^-1 ^and mmol/(h·gDW), that is, mmol per hour per gram dry weight of *M. tuberculosis*. Equation 1 did not include a cellular death rate because we simulated bacterial growth during exponential stage and early stationary stage conditions, where cellular growth plays a more dominant role than cellular death. Previous studies under similar growth conditions, which also did not explicitly include a death rate term, obtained simulation results that were consistent with experimental data [25,30].

The biomass growth rate *μ *and the uptake rate of the limiting substrate *v*_*C *_are determined, for a given time point, by performing a FBA of the metabolic network for a given inhibitor concentration [*I*] and an upper limit constraint on the limiting substrate uptake rate . We formally introduced a notation to indicate the growth rate *μ *and the uptake rate *v*_*C *_outputs of a FBA of a metabolic network for a given set of input conditions [*I*] and  as:

(3)

We employed the Michaelis-Menten kinetic model [25] to estimate the upper limit of the substrate uptake rate:

(4)

where *V*_*m *_denotes the maximum initial rate of substrate uptake and *K*_*m *_represents the Michaelis-Menten rate constant. Moreover, we linked the experimental readout, in this case the optical density (*OD*) under 600-nm-wavelength light [46,53], to the cell concentration [*X*] by:

(5)

where K denotes a proportionality constant. Other experimental readouts can be similarly accounted for.

### Sensitivity analysis of parameter values

The presence of a number of parameters in our mathematical framework warranted a sensitivity analysis as to how the assigned parameter values affected the final computational results. We used two different metrics to ascertain parameter sensitivity. In the first analysis, we gauged the variation in the results by separately setting each one of the parameter values to reasonable lower and upper bounds [10], in this case, ± 50% of the chosen parameter values. In the second analysis, we computed the sensitivity coefficient for each of the parameters. This coefficient provides a measure of the dependency between the computed results and the corresponding parameter. If *OD *represents the cell concentration expressed as optical density under 600-nm-wavelength light and *p *represents the parameter analyzed for sensitivity, the sensitivity coefficient  is defined as follows [59,60]:

(6)

Other observables, different from *OD*, can be substituted for in Eq. 6. To numerically calculate the sensitivity coefficient  for a parameter *p*, we started from ∂*p *= +0.5*p *and repeated the process by reducing ∂*p *and calculating the sensitivity coefficient until  converged, that is, until successive values of ∂*p *yielded the same . We then repeated the process starting from ∂*p *= -0.5*p *until convergence. In the calculation performed here, both processes converged to the same numerical value.

To address the different types of parameters in our framework, we classified the modeled parameters into four groups: *group I *included parameters obtained from the literature, *group II *included those determined by matching experimental data, *group III *included those assumed to be derived from other parameters, and *group IV *included those that, by definition, were directly determined once the other parameters were defined. During the sensitivity analysis of the parameters in *groups I*, *II*, and *III*, we calculated dose-response curves while increasing and decreasing each parameter by 50% (except for those whose values cannot exceed one) and sensitivity coefficients spanning three orders of magnitude in inhibitor concentration. Although the parameters in *group III *were assumed to be derived from the parameters in *groups I *and *II*, during the sensitivity analysis for the first two groups we held the parameter values in *group III *fixed. Also, because the parameters in *group IV *were dependent on other parameters and their values changed as we performed sensitivity analysis on these independent parameters, we did not perform analysis for this group.

## Results

### Modeling cell growth inhibition by 3-NP

#### Nutrient-poor environment and in-vivo growth

**Figure 2:**
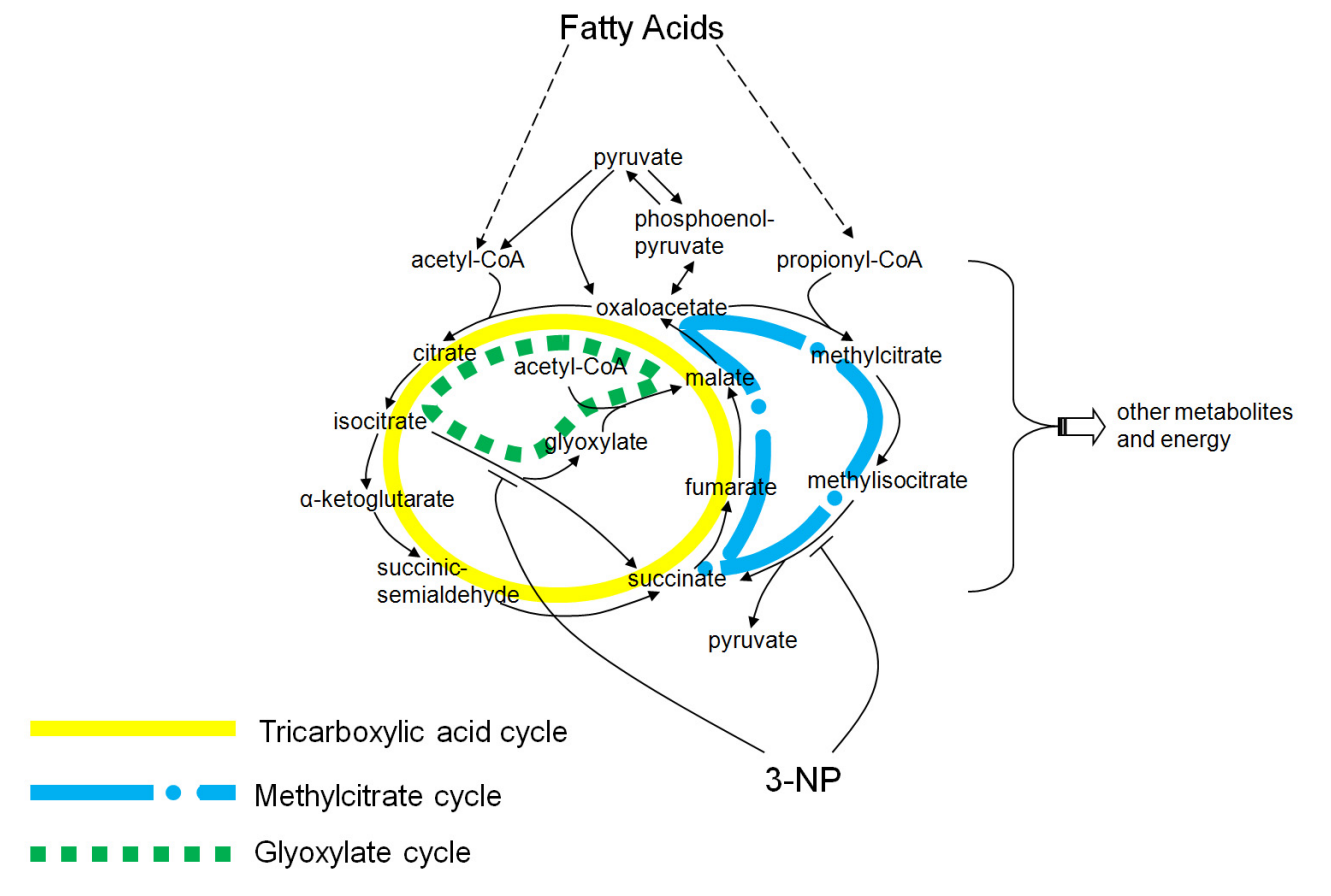
**The pathways for utilizing fatty acids, showing the target reactions of the 3-nitropropionate (3-NP) inhibitor**. The fatty acid pathways include the tricarboxylic acid cycle marked by the solid line (oxaloacetate → isocitrate → α-ketoglutarate → succinate → malate → oxaloacetate), the glyoxylate cycle marked by the dashed line (oxaloacetate → isocitrate → glyoxylate → malate → oxaloacetate), and the methylcitrate cycle marked by the dash-dotted line (oxaloacetate → methylcitrate → methylisocitrate → succinate → malate → oxaloacetate). 3-NP inhibits the enzymes that catalyze the reactions involved in converting isocitrate and methylisocitrate to succinate. CoA = coenzyme A.

*M. tuberculosis *faces a hostile and harsh environment upon infecting a mouse or a human host. The invasion of *M. tuberculosis *stimulates the activation of host immunity systems initiated by the release of macrophages that ingest pathogen cells. Macrophage-ingested cells are contained in phagosomes where they confront high pH, antibacterial reactive oxygen and nitrogen, and the lack of carbohydrates [38]. Because of the lack of carbohydrate nutrients, the growth and survival of *M. tuberculosis *requires fatty acids as the principal carbon source [45]. Figure 2 sketches out the metabolic pathways that represent a portion of the much larger metabolic network of the organism (not shown) through which fatty acids are utilized by the bacterium. Fatty acids are converted into acetyl-coenzyme A (CoA) and propionyl-CoA through β-oxidation. The conversion of acetyl-CoA to other metabolites, like pyruvate, requires the availability of the glyoxylate cycle (dashed line in Figure 2) that converts isocitrate to malate through a glyoxylate intermediate. This pathway is an attractive drug target because it appears to be absent in mammalian cells [46]. The metabolism of propionyl-CoA goes through the methylcitrate cycle (dash-dotted line in Figure 2), which converts oxaloacetate, through methylcitrate, to produce succinate.

Figure 2 shows where the 3-NP inhibitor affects two key reactions in the glyoxylate and methylcitrate cycles [46]. These metabolic reactions are catalyzed by the enzymes isocitrate lyase 1 (ICL1) and isocitrate lyase 2 (ICL2), expressed by the *icl1 *and *icl2 *genes, respectively. The two metabolic reactions catalyzed by these enzymes convert a citrate substrate into succinate and a by-product [44,46]. The inhibitor-targeted metabolic reactions of isocitrate lyase (ICL) and methylisocitrate lyase (MCL) are defined as:

(7)

(8)

It has been experimentally shown that 3-NP inhibits the growth of *M. tuberculosis *in fatty acid media and in mouse macrophage cells [46]. Medium containing propionate (C_2_H_5_COO^-^) an odd-chain fatty acid as the main carbon source was used in an *in vitro *experimental model system to investigate *in vivo *inhibition [46]. Here, we similarly modeled the inhibition effect of 3-NP on the *M. tuberculosis *growth on *in vitro *media as a prelude to the more complicated study of modeling drug effects in an *in vivo *host-cell environment.

#### Experiment-specific mathematical framework

Experiments show that 3-NP inhibits the growth of *M. tuberculosis *when propionate is the major carbon source in the medium used to grow the bacterium [46]. To quantitatively model this inhibition effect, we needed to assemble the mathematical framework that was specific to this medium and inhibition process. The terminology "inhibitor," "target reaction," and "substrate" in Figure 1 refer to 3-NP, the reactions ICL and MCL, and propionate, respectively. The appropriate specifications needed for the inhibition model, metabolic network, and population growth model are described below.

##### 3-NP inhibition model

We used the previously developed kinetic equation for the 3-NP-inhibited ICL reaction [21] to relate inhibitor concentration to the flux ratio of the reactants. This assumed that changes in intracellular enzyme and metabolite concentrations are relatively unaffected by the presence of 3-NP and that the ICL reaction is irreversible [42]. Accordingly, the inhibition model relating the concentration of the 3-NP inhibitor [3-NP] to the resulting flux ratio *f*_*ICL*_([3-NP]) of the ICL reaction was given by:

(9)

where *v*_*ICL *_and  denote the inhibitor and inhibitor-free reaction fluxes, respectively, *w*_*ICL1 *_and *w*_*ICL2 *_represent the fractions of the overall inhibitor-free ICL reaction flux for the ICL1- and ICL2-catalyzed reaction components, respectively, *SUC *denotes the succinate substrate, [*SUC*] indicate its concentration, and *K*_3-*NP*, *ICL1*_, *K*_3-*NP*, *ICL2*_, *K*_*SUC*, *ICL1*_, and *K*_*SUC*, *ICL2 *_denote Michaelis constants [25].

The parameter values for this model can be partially found in the literature, but some of them need to be calculated or fitted to match experimental conditions. The values of *w*_*ICL1 *_and *w*_*ICL2 *_were estimated from the known rate constants for the ICL reaction catalyzed by the ICL1 and the ICL2 enzymes, respectively. Thus, given the reaction rates of 5.24 s^-1 ^and 1.38 s^-1 ^for ICL1 and ICL2, respectively [44], the fractions of *w*_*ICL1 *_and *w*_*ICL2 *_were estimated to be 0.79 and 0.21, respectively. The experimental values employed for the succinate concentration were used to set [*SUC*] to 2.464 mM [21]. Likewise, we directly used the experimentally determined values of 0.003 mM and 0.11 mM for *K*_3-*NP*, *ICL1 *_and *K*_3-*NP*, *ICL2*_, respectively [21,61]. The value for *K*_*SUC*, *ICL1 *_was obtained by using the 3-NP specific mathematical framework to match the experimental cell concentration at a specific 3-NP concentration of 0.025 mM (this is described in the next subsection "Obtaining Undetermined Parameter Values"). Based on the experimentally measured range of *K*_*SUC*, *ICL2 *_values [21], we set this parameter to 10 × *K*_*SUC*, *ICL1*_.

Because the kinetic equation for the MCL reaction is not available, and based on the strong similarity between the mechanisms of catalysis and 3-NP inhibition of the MCL and the ICL reactions, we assumed it had the same form as the ICL reaction:

(10)

The variables and parameters in this equation have similar meaning to those given in Eq. 9. The unavailable values for *K*_3-*NP*, *MCL*1_, *K*_3-*NP*, *MCL*2_, *K*_*SUC*, *MCL*1_, and *K*_*SUC*, *MCL*2 _were set to the same corresponding values used in the ICL model. The fractions *w*_*MCL*1 _and *w*_*MCL*2 _were set to 0.999 and 0.001, respectively, as the associated rate constants of the MCL reaction for the ICL1 and the ICL2 enzymes are 1.25 s^-1 ^and <10^-3 ^s^-1^, respectively [44].

##### Metabolic network considerations

The metabolic network model must take into account the appropriate substrate uptake and target reaction constraints based on the experimental setting [46]. Here, we first focus on substrate uptake in propionate medium used in the experiment. The propionate uptake was constrained based on the propionate concentration in the medium. Because setting the glycerol uptake rate to zero would have caused the biomass growth rate to be zero [42], we set this uptake rate to a very small value, 0.001 mmol/(h·gDW). In *iNJ*661 [42], glycerol is a biomass component, but there is no pathway to synthesize glycerol, necessitating the addition of a small amount of glycerol uptake to the metabolic network. The uptake rates of other carbon sources, like glucose, were set to zero. Other necessary substrate uptake rates, including phosphate, sulfate, ferric iron, ammonium, and oxygen, were left unconstrained. In the absence of a 3-NP inhibitor in the medium, the fluxes of the ICL and MCL reactions in Eqs. 6 and 7 were unconstrained and the inhibitor-free reaction fluxes  and  were obtained from a FBA calculation. When 3-NP was present, the ICL (and MCL) reaction fluxes were constrained to be no more than the product of the flux ratios of *f*_*ICL*_([3-NP]) (or *f*_*MCL*_([3-NP])), determined from the inhibition model, and the inhibitor-free fluxes *f*_*ICL*_ (or *f*_*MCL*_). We constrained the target reaction fluxes to upper bounds instead of fixing them to specific values in order to avoid an artificial coupling of fluxes. For example, in the case of 3-NP, which inhibits both ICL and MCL reactions, the resultant fluxes may be different and may not be equal to but lower than the constraints. When the *M. tuberculosis *deletion mutant *Δicl1Δicl2 *was studied, the fluxes associated with the ICL and MCL reactions were set to zero [11,12]. The developed metabolic network, including the constrained substrate uptake rates, is available in Systems Biology Makeup Language (SBML) format (see Additional file 2).

##### Experimental population growth model

In the experimental study of the 3-NP inhibitor, cell concentrations are monitored at different time points during a 16-day growth experiment in propionate medium with and without inhibitor [46]. We can computationally obtain the same growth curves by consistently solving Eqs. 1-5 with the appropriate specific experimental conditions. For this set of equations, propionate is the limiting substrate *C*, *v*_*C *_is the propionate uptake rate, and  is the upper limit constraint on the propionate uptake rate. Because the optical density *OD *was used as the readout of the experiments, we did not provide absolute values for the cell concentration [*X*] [46]. By defining [*C'*] = [*C*]/*K *and  = *K*_*m*_/*K*, Eqs. 1-5 can be written as:

(11)

(12)

(13)

(14)

The initial values for *OD *were taken directly from the experimental data [46]. The population growth model defined by Eqs. 11-14 were then iteratively solved by using the results generated from the FBA of the metabolic network.

#### Obtaining undetermined parameter values

All parameters needed to calculate cellular growth and growth inhibition from the mathematical framework in Figure 1 have not been experimentally determined. However, we could use the combined formalism of the three models to self-consistently determine the unknown parameter values. In particular, we needed to estimate values for the initial propionate concentration [*C'*] (*t = 0*), the maximum initial propionate uptake rate *V*_*m*_, the Michaelis-Menten rate constant for the propionate uptake  in Eq. 14, and *K*_*SUC*, *ICL1 *_in Eq. 9.

**Figure 3:**
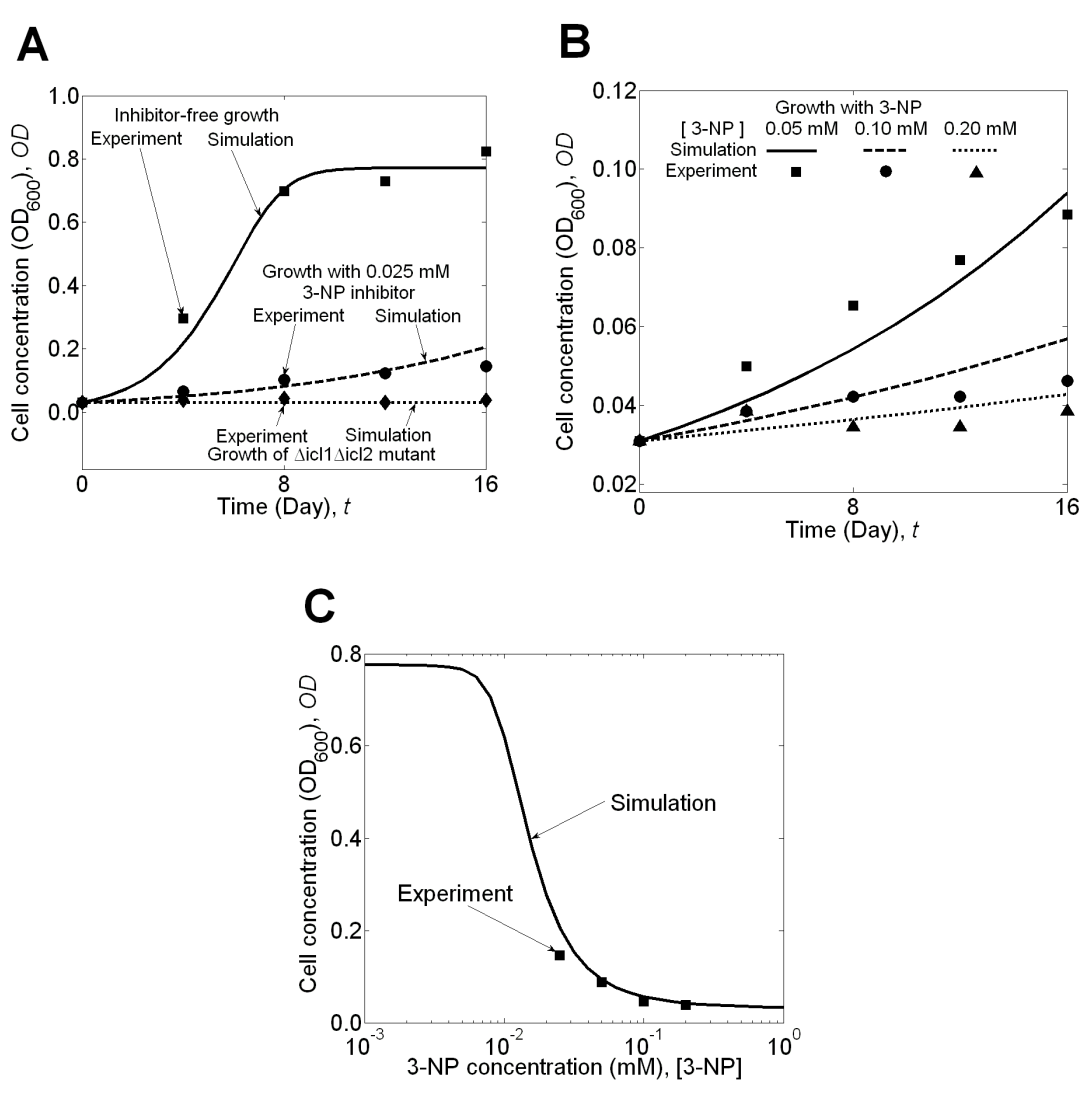
**Results from the mathematical framework used to study the inhibitory effects of 3-nitropropionate (3-NP)**. **(A) **Cell concentration, expressed in units of optical density at 600-nm-wavelength light (OD_600_), of *Mycobacterium tuberculosis *in inhibitor-free medium (*solid line*), in medium with 0.025 mM 3-NP (*dashed line*), and cell concentrations of the *Δicl1 Δicl2 *mutant bacterium (*dotted line*) obtained from our calculation using the described mathematical framework and compared to the corresponding experimental results [46]; **(B) **The calculated cell concentration, expressed as OD_600_, of *M. tuberculosis *is shown as a function of time for different 3-NP inhibitor concentrations and compared to the corresponding experimental data [46]; and **(C) **The calculated cell concentration, expressed as OD_600_, of *M. tuberculosis *after a 16-day growth period as a function of 3-NP inhibitor concentration compared to experimental values [46].

We first determined the values of three of these four parameters by matching the inhibitor-free growth curve of *M. tuberculosis*. We systematically manipulated the values for [*C'*] (*t = 0*), *V*_*m*_, and  to reproduce the experimental cell concentrations (see Additional file 1: Section S2). Figure 3A (solid line) shows the match between simulation results and experimental data of inhibitor-free growth when [*C'*] (*t = 0*), *V*_*m*_, and  were set to 40 mmol/gDW, 2 mmol/(h·gDW), and 30 mmol/gDW, respectively. Next, we used the experimental cell concentration data for *M. tuberculosis *in propionate medium containing 0.025 mM 3-NP inhibitor to estimate the value of the fourth and last unknown parameter, *K*_*SUC*, *ICL1*_. This was achieved by mathematically varying the value of *K*_*SUC*, *ICL1 *_until we obtained close agreement between experimental and predicted growth data, as shown in Figure 3A (dashed line). This process set the value of *K*_*SUC*, *ICL1 *_to 1.5 mM (see Additional file 1: Section S2).

#### Verification of essentiality of the target reactions

A prerequisite for a good inhibitor is that its target is essential for the survival and homeostasis of the bacterium. In the experimental study, genes *icl1 *and *icl2 *whose products catalyze the reactions ICL and MCL, respectively, are deleted from wild-type *M. tuberculosis*. Figure 3A shows that in the experiment the resultant deletion mutant *Δicl1Δicl2 *exhibits a lack of growth in propionate medium [46]. We used the mathematical framework to verify that the two 3-NP-inhibited reactions, ICL and MCL, are necessary for the growth of *M. tuberculosis *in this medium. This was achieved by setting the fluxes associated with these reactions to zero (see Additional file 1: Section S3), leading to a model that predicts complete lack of bacterial growth in the absence of these reactions (dotted line in Figure 3A).

#### Growth predictions

As described above in "Obtaining Undetermined Parameter Values," we used the experimentally determined cell concentration growth at the lowest (0.025 mM) of the four measured inhibitor concentrations 0.025, 0.050, 0.100, and 0.200 mM [53] to determine the value of *K*_*SUC*, *ICL1*_. With the values of the rest of parameters established, we then used the mathematical framework to predict the growth of *M. tuberculosis *at the other three 3-NP inhibitor concentrations (0.050, 0.100, and 0.200 mM) and compared the results to the experimental values (see Additional file 1: Section S4). Figure 3B shows that, for each inhibitor concentration, the predicted and the experimental cell concentrations were, overall, in good agreement with each other. However, Figure 3B also shows that the simulation results may over- or under-predict the experimental data. This was due to the maximization of biomass growth rate and also by not including an explicit cellular death rate, assumptions that are reasonable for modeling growth in exponential and early stationary stages (see section "Development of the Mathematical Framework"). Thus, implementation of our modeling framework under different conditions or for very long time periods may cause mismatches when simulating cellular growth. Figure 3C shows that the simulated dose-response curve, which links the 3-NP concentrations to the cell concentrations after a 16-day growth of *M. tuberculosis*, provided accurate predictions matching the experimental results.

We used linear regression [62] to evaluate the fitness between the simulation results and the experimental data. For the 30 experimental data points shown in Figures 3A and 3B, we used our framework to obtain the corresponding simulated values. A linear regression of the data yielded a slope (1.0008) and intercept (0.0001) close to one and zero, respectively. The coefficient of determination (*R*^2^) was 0.9867, indicating a strong and significant correlation (*P *value = 8.2050 × 10^-28^) between the simulated values and experimental data.

The benefit of a quantitative predictive model lies both in the ability to rapidly make predictions once the model is properly parameterized and the additional insights gained in the mechanisms underlying the experimental observables. With a model, we can accurately predict dose-response curves in less than one hour, and the mathematical framework can provide information that cannot be directly obtained from an experiment. For example, we knew that 3-NP inhibits both the ICL and the MCL reactions defined in Eqs. 6 and 7, respectively. However, the degree to which each reaction slows down growth was not known. This question is experimentally difficult to ascertain, since the two reactions are catalyzed by the same enzyme. Theoretically, we can use the developed formalism to answer this question by allowing 3-NP to inhibit only one reaction. The calculated growth of *M. tuberculosis *in medium with 0.025, 0.050, 0.100, and 0.200 mM 3-NP was very similar to the inhibitor-free growth when only the ICL reaction was affected by the inhibitor. However, when we assumed that 3-NP only inhibited the MCL reaction, the calculated growth was virtually the same as the results in Figure 3A (dashed line) and Figure 3B. Therefore, the simulations suggest that it was primarily the inhibitory effect of 3-NP on the MCL reaction that limited *M. tuberculosis *growth. This observation is compatible with the metabolism of odd- and even-chain fatty acids. Both the ICL and the MCL reactions are necessary steps in transferring extracellular carbon atoms to intracellular metabolites and obtaining energy from fatty acids. However, these reactions differ in that the ICL reaction is used for even-chain fatty acids, the MCL reaction is used for odd-chain fatty acid with three carbon atoms such as propionate, and longer odd-chain fatty acids require both reactions [44]. Because in the studied medium propionate is the major carbon source, the inhibition of the MCL reaction is key to the inhibitory effect of 3-NP.

#### Sensitivity analysis of parameter values

**Figure 4:**
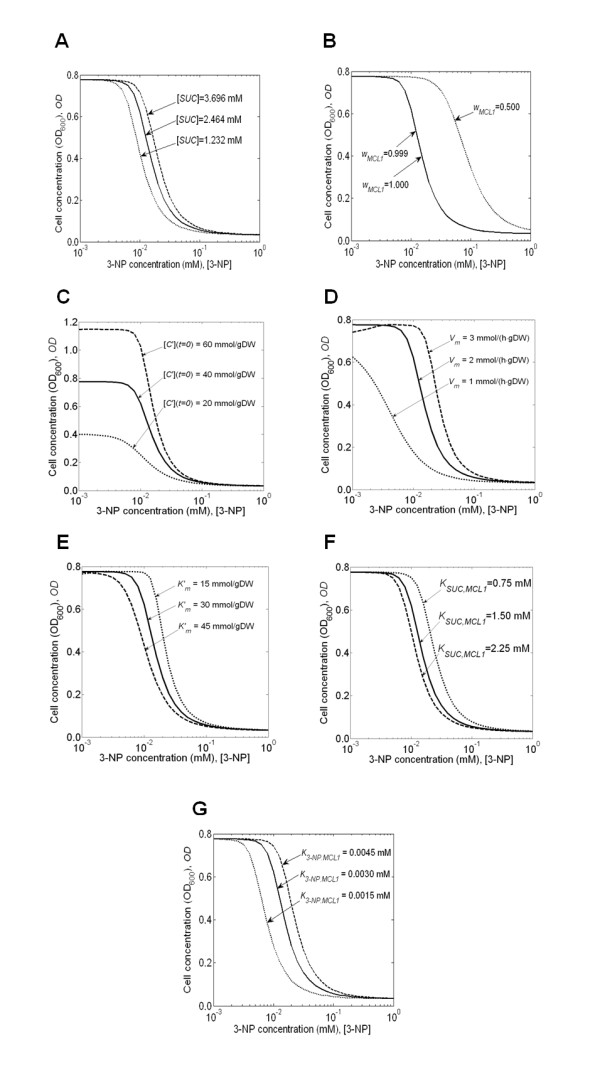
**The influence of the parameter values on the calculated dose-response curve**. Sensitivity analysis of the calculated cell concentration, expressed in units of optical density at 600-nm-wavelength light (OD_600_), of *Mycobacterium tuberculosis *after a 16-day growth period as a function of 3-nitropropionate (3-NP) concentration. The analysis was performed for the parameters set to their original values (*solid lines*), those values increased by 50% (*dotted lines*). **(A-G) **Sensitivity of the dose-response curves for variations in the values of the **(A) **succinate concentration [*SUC*]; **(B) **ICL1-catalyzed fraction of the overall inhibitor-free MCL reaction flux *w*_*MCL1*_; **(C) **initial propionate concentration [*C'*] (*t *= 0); **(D) **maximum initial propionate uptake rate *V*_*m*_; **(E) **Michaelis-Menten rate constant for the propionate uptake ; **(F) **Michaelis-Menten rate constant *K*_*SUC,MCL1*_; and **(G) **Michaelis-Menten rate constant *K*_*3-NP,MCL1*_.

Table 1 shows the four groups of 16 parameters used to construct the 3-NP inhibition model. Figure 4 shows the extent of the computed dose-response curve variations for the seven parameters that materially affected the results: [*SUC*] and *w*_*MCL1 *_in *group I*; [*C'*] (*t = 0*), *V*_*m*_, and  in *group II*, and *K*_*SUC*, *MCL1 *_and *K*_3-*NP*, *MCL1 *_in *group III*. Figure 4A shows that, compared with the other six parameters whose variations influenced the dose-response curve, the succinate concentration [*SUC*] had relatively a small effect, suggesting that it was reasonable to assign [*SUC*] a constant value in the model. Figure 4B shows that direct variations of *w*_*MCL1 *_introduced relatively large variations in the calculated dose response. Because this parameter was originally derived from a relative ratio of experimentally determined rate constants, the ± 50% variations of *w*_*MCL1 *_greatly exaggerated plausible experimental errors. For this reason, the sensitivity coefficient analysis below gives a more realistic measure of the importance of this experimentally determined parameter. Changes in the initial propionate concentration [*C'*] (*t *= 0) (Figure 4C) and in the maximum initial propionate uptake rate *V*_*m *_(Figure 4D) had large effects on the dose-response curves because directly adding or subtracting nutrients and allowing for different nutrient uptake rates directly affected cellular growth. Moreover, Figure 4D shows an additional non-linear effect in the dose-response curve for the largest uptake rate [*V*_*m *_= 3 mmol/(h·gDW)] for low inhibitor concentrations (≤0.003 mM). For large values of *V*_*m*_, the effective biomass production per unit of propionate uptake became lower and pushed the bacterium into a growth regime where propionate could not be efficiently used. Hence, for low inhibitor concentrations, the cell concentrations at the largest *V*_*m *_(dashed line in Figure 4D) were lower than those at the original *V*_*m *_(solid line in Figure 4D). Figures 4E, 4F, and 4G show that independent variations of , *K*_*SUC*, *MCL*1_, and *K*_3-*NP*, *MCL1 *_induced a similar magnitude change in the calculated dose-response curves. These parameters show a similar range of variation, although both  and *K*_*SUC*, *MCL1 *_were ultimately derived from matching experimental data, whereas *K*_3-*NP*, *MCL1 *_was, in effect, an experimentally determined parameter.

**Table 1:** Model parameters for cell growth inhibition by 3-NP.

**Group**	**Parameter**	**Model**	**Equation**	**Source of the value**
I	*w*_*ICL1*_	Inhibition Model	9	Set to be 0.790 from [44]
	
	[*SUC*]	Inhibition Model	9	Set to be 2.464 mM from [21]
	
	*K*_3-*NP*, *ICL1*_	Inhibition Model	9	Set to be 0.003 mM from [21,61]
	
	*K*_3-*NP*, *ICL2*_	Inhibition Model	9	Set to be 0.110 mM from [21,61]
	
	*w*_*MCL*1_	Inhibition Model	10	Set to be 0.999 from [44]

II	[*C'*] (*t *= 0)	Population Growth Model	14	Obtained by matching experimental cell growth data
		
	*V*_*m*_	Population Growth Model	14	
		
		Population Growth Model	14	
		
	*K*_*SUC*, *ICL1*_	Inhibition Model	9	

III	*K*_*SUC*, *ICL2*_	Inhibition Model	9	Assumed (based on [21]) to be 10 × *K*_*SUC*, *ICL1*_
	
	*K*_*SUC*, *MCL*1_	Inhibition Model	10	Assumed to be equal to *K*_*SUC*, *ICL1*_
	
	*K*_*SUC*, *MCL*2_	Inhibition Model	10	Assumed to be equal to *K*_*SUC*, *ICL2*_
	
	*K*_3-*NP*, *MCL*1_	Inhibition Model	10	Assumed to be equal to *K*_3-*NP*, *ICL1*_
	
	*K*_3-*NP*, *MCL*2_	Inhibition Model	10	Assumed to be equal to *K*_3-*NP*, *ICL2*_

IV	*w*_*ICL2*_	Inhibition Model	9	Equal to 1-*w*_*ICL1*_
	
	*w*_*MCL*2_	Inhibition Model	10	Equal to 1-*w*_*MCL1*_

The parameter values were grouped into four groups depending on their origin: *group I *parameters contained those obtained from the literature; *group II *parameters were obtained by matching experimental growth data; *group III *parameters were assumed to be related to the parameters of the first two groups; and *group IV *parameters are, by definition, determined once the group I parameters were defined.

The other parameters of the model had no effect on the calculated dose-response curves. The parameters *w*_*ICL1*_, *K*_3-*NP*, *ICL1*_, *K*_3-*NP*, *ICL2*_, *K*_*SUC*, *ICL1*_, and *K*_*SUC*, *ICL2*_, used in the definition of the 3-NP inhibition model in Eq. 8, did not affect the results because 3-NP primarily inhibited growth through the MCL reaction and not the ICL reaction (see previous subsection "Growth Predictions"). Similarly, *K*_*SUC*, *MCL*2 _and *K*_3-*NP*, *MCL*2_, relating to the ICL2 enzyme in the second term of Eq. 10, did not affect the calculated dose-response curves.

Table 2 shows the calculated sensitivity coefficient  for each model parameter at different 3-NP concentrations. The sensitivity coefficients provided a quantitative measure, allowing us to gauge the relative importance of each parameter. Interestingly, although the dose-response curves in Figure 4 indicated that (except for *w*_*MCL*1_) the absolute changes of cell concentration around 10^-1 ^mM 3-NP were small, the values in Table 2 showed that the calculated sensitivity coefficients at this concentration were not necessarily smaller than those at other inhibitor concentrations.

**Table 2:** Sensitivity coefficients for the parameters in modeling 3-NP inhibition.

**Parameter *p***	**Sensitivity Coefficient as a Function of [3-NP]**			
	
	**0.001 mM**	**0.01 mM**	**0.1 mM**	**1 mM**
*w*_*ICL1*_	0.0000	0.0000	0.0000	0.0000

[*SUC*]	0.0004	0.4657	0.3380	0.0394

*K*_3-*NP*, *ICL1*_	0.0000	0.0000	0.0000	0.0000

*K*_3-*NP*, *ICL2*_	0.0000	0.0000	0.0000	0.0000

*w*_*MCL*1_	-0.0007	-1.4741	-3.8957	-0.8557

[*C' *] (*t *= 0)	0.9609	1.2746	0.2596	0.0278

*V*_*m*_	0.0060	1.3459	0.5908	0.0646

	-0.0032	-0.7049	-0.2555	-0.0277

*K*_*SUC*, *ICL1*_	0.0000	0.0000	0.0000	0.0000

*K*_*SUC*, *ICL2*_	0.0000	0.0000	0.0000	0.0000

*K*_*SUC*, *MCL*1_	-0.0004	-0.4657	-0.3377	-0.0393

*K*_*SUC*, *MCL*2_	-0.0000	-0.0000	-0.0003	-0.0001

*K*_3-*NP*, *MCL*1_	0.0007	0.7492	0.5432	0.0632

*K*_3-*NP*, *MCL*2_	0.0000	0.0002	0.0020	0.0008

The sensitivity coefficient  was calculated from a numerical estimate of , where *OD *is the cell concentration expressed in units of optical density at 600-nm-wavelength light and *p *represents the analyzed parameter.

Although our framework is capable of modeling growth inhibition as a function of inhibitor concentration, it would still be desirable to reduce the uncertainty in the model parameters by directly obtaining accurate parameter values from experimental studies. The predictive power of our model could be further refined if the relationship between succinate concentrations [*SUC*] and the fluxes of the ICL and MCL reactions inside *M. tuberculosis *cells could be ascertained experimentally. This could be done by jointly measuring intracellular metabolite concentrations and metabolic fluxes, as recently done in a study of *E. coli *metabolism [63]. Similarly, values for *K*_3-*NP*, *MCL1 *_and *K*_*SUC*, *MCL1 *_could be obtained directly from enzyme kinetic experiments [64].

### Modeling cell growth inhibition by sAMS

#### The importance of iron sequestration

As a response to the invasion of *M. tuberculosis*, the host immune system reduces the iron levels in pathogen-infected environments by means of iron-binding proteins [48]. *M. tuberculosis *responds to the changing environment by synthesizing and secreting mycobactin, which has an extremely high iron affinity and helps the pathogen obtain iron from host proteins [53]. The synthesis of mycobactin is thus an essential step for the survival and growth of *M. tuberculosis *inside the host and provides a potential drug target with broad anti-bacterial applicability.

**Figure 5:**
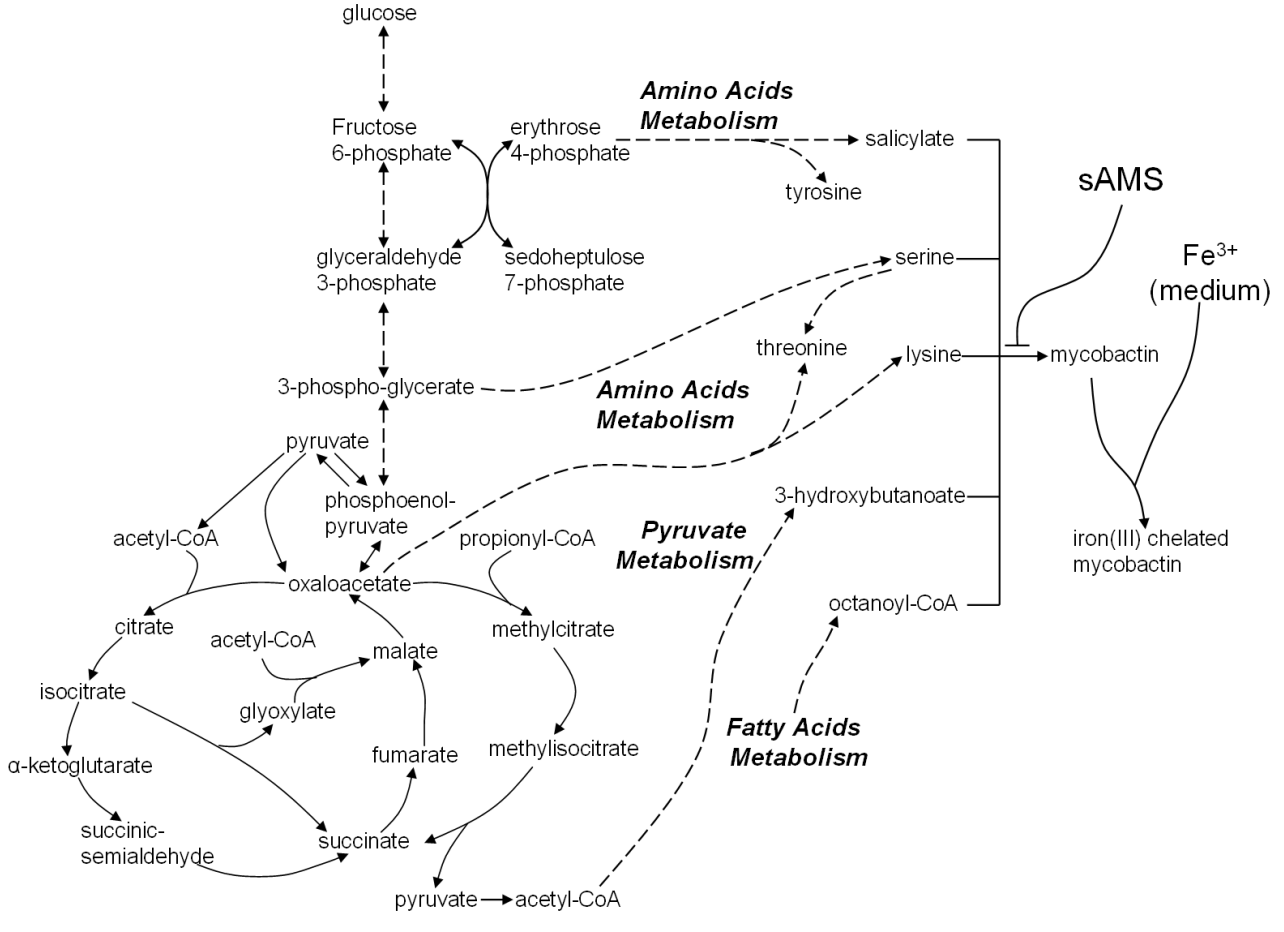
**The metabolic pathways involved in the mycobactin synthesis and subsequent iron uptake**. The target reaction for the 5'-*O*-(*N*-salicylsulfamoyl) adenosine (sAMS) inhibitor is indicated at the top right. The connection to the metabolic pathways inhibited by 3-nitropropionate (3-NP) in Figure 2 is shown at the lower left. Note that only parts of the metabolic network are indicated in the figure. The entire network consists of 830 metabolites and 1,031 reactions.

Figure 5 outlines the metabolic pathways extracted from the complete metabolic network that are required for mycobactin synthesis. These pathways include the tricarboxylic acid cycle, the glyoxylate cycle, and the methylcitrate cycle outlined in Figure 2, and show that the related metabolites and additional pathways, such as the amino acids metabolism, are used to produce mycobactin. Conversely, sAMS is an inhibitor that targets mycobactin synthesis [53]. Although the effects of this inhibitor in the host environment have not yet been reported, its inhibition of the *in vitro *growth of *M. tuberculosis *in an iron-deficient medium, matching the host-cell environment [53], points to its potential therapeutic value.

#### Experiment-specific mathematical framework

To implement the mathematical framework outlined in Figure 1, we customized the models to account for the action of sAMS ("inhibitor") on the synthesis of mycobactin ("target reaction"). Glycerol, alanine, and salts in the medium used in the experimental studies of this inhibitor [53] were modeled as "substrates." The appropriate specifications needed for the inhibition model, the metabolic network, and the population growth model are described below.

##### sAMS inhibition model

sAMS inhibits the enzyme salicyl-AMP ligase (MbtA; encoded by the gene Rv2384) that catalyzes the synthesis of mycobactin and is characterized as a tight-binding inhibitor. Therefore, Morrison's equation can be used to specify the inhibition model that relates the concentration of the sAMS inhibitor [sAMS] to the flux ratio of the mycobactin synthesis reaction *f*_*MS*_, considering the concentration of the MbtA enzyme [*E*] as one parameter [53]:

(15)

where *v*_*MS *_and  denote the flux of the mycobactin synthesis reaction in the presence and in the absence of the sAMS inhibitor, respectively, and  is an "apparent" reaction rate constant whose value is 0.7 nM [53]. To study the effect of the inhibitor on the isolated MbtA enzyme, we used the same values taken in the *in vitro *experimental assay and set [*E*] = 20 nM [53]. For intracellular environment studies, the value of [*E*] is unknown but can be inferred, as described below (see subsection "Obtaining Undetermined Parameter Values").

##### Metabolic network considerations

To duplicate the experimental conditions of the sAMS inhibitor study, we constrained the substrate uptake based on the medium used in the experiment [53]. The medium contained glycerol, alanine, salts, and Tween (GAST) and the amount of added iron defined the medium condition as iron-deficient or sufficient [53]. The Tween component of the medium acts as a detergent in the experimental system and was not included as a substrate in the metabolic network. Glycerol and alanine are major carbon sources whose uptake rates were constrained to be no more than 1 mmol/(h·gDW) [42]. The uptake rates of the salts, oxygen, and water were unconstrained in the metabolic network. We also modified the biomass composition for iron-sufficient medium by changing the metabolite "iron(III) chelated carboxymycobactin T" into iron(III), since mycobactin synthesis and chelation were absent in this medium. The constraint placed on the target reaction followed the approaches used in the 3-NP study. Thus, when sAMS was present, the reaction flux was constrained to be no more than the product of *f*_*MS *_and . The developed metabolic network, including the constrained substrate uptake rates, is available in SBML format (see Additional file 3).

##### Experimental population growth model

The experimental study of the sAMS inhibitor reports the relative cell concentrations, which represent the ratios of cell concentrations in the presence to the absence of the inhibitor after eight days of growth [53]. The experimental data show no apparent lag time between the start of cell growth and the onset of exponential growth [46,49]. Moreover, because cell growth usually does not enter into a stationary stage during the first eight days [46,49], we assumed an exponential growth in which the growth rate and the substrate uptake rate were nearly constant. Therefore, the ODE for the cell concentration [*X*] in Eq. 1 was directly integrated to give:

(16)

where [*X*_*t *= 0_] denotes the initial cell concentration, which is the same whether sAMS was present or not. The relative cell concentration *R*_*C *_after eight days was obtained as:

(17)

where [*X*^0^] denotes the inhibitor-free cell concentration, *t *is set to eight days, and the inhibitor-present biomass growth rate *μ *and the inhibitor-free biomass growth rate *μ*^0 ^were inferred from the metabolic network using FBA.

#### Obtaining undetermined parameter values

Among the parameters needed to study the inhibitory effect of sAMS on *M. tuberculosis *growth, only the intracellular MbtA-enzyme concentration [*E*] in Eq. 15 was not readily available from the experimental data. To obtain this parameter value, we selected a relative cell concentration *R*_*C *_of 0.49 at a sAMS concentration [sAMS] of 1.7 μM from the growth-inhibition experiment in iron-deficient medium [53] and varied the value of [*E*] in Eq. 15 until our framework reproduced this *R*_*C *_value. This point was selected because 0.49 is close to the mid-range of *R*_*C *_values, 0 ≤ *R*_*C *_≤ 1. After trying different values for [*E*], we found that [*E*] = 40 μM yielded good agreement between the calculated (0.47) and selected (0.49) relative cell concentrations (see Additional file 1: Section S5).

#### Verification of essentiality of the target reactions

To meet the minimum requirements of an inhibitor, sAMS needs to target a reaction that is essential for cellular survival and function. From the experimental analysis, we noted that, as the sAMS concentration increases to a large value (~10^3 ^μM), the measured relative cell concentration becomes close to zero [53]. Similarly, our mathematical framework needs to be capable of reproducing the essentiality of the targeted reaction, mycobactin synthesis in the presence of sAMS, for cellular growth of *M. tuberculosis *in iron-deficient GAST medium. Thus, we set the flux of the mycobactin synthesis reaction to zero, which, as expected, yielded a relative cell concentration *R*_*C *_of zero (see Additional file 1: Section S6). This suggests that the essentiality of the mycobactin synthesis reaction was duplicated in our framework.

#### Growth Predictions

**Figure 6:**
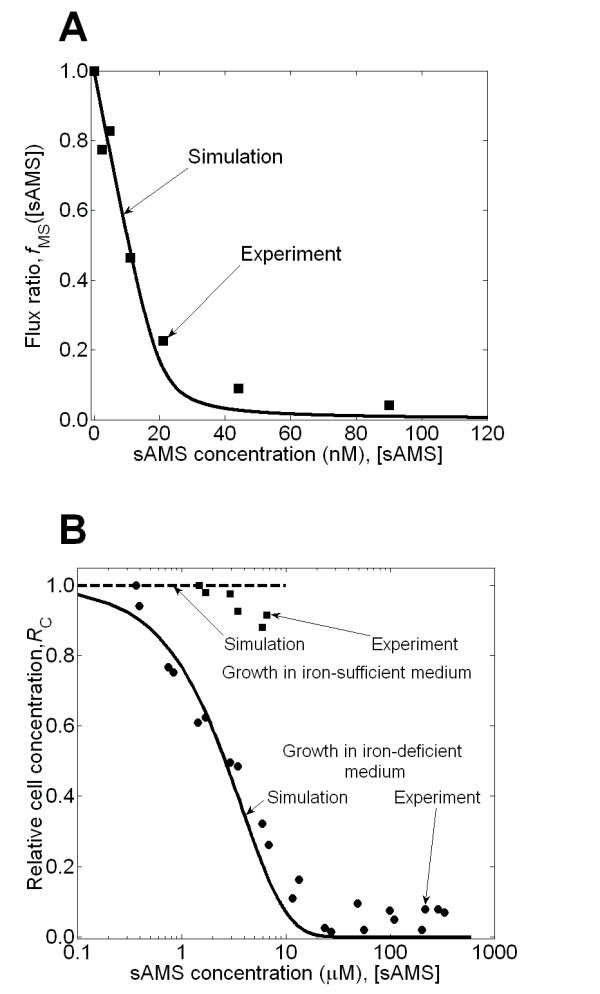
**Results for the study of the inhibitory effects of 5'-*O*-(*N*-salicylsulfamoyl) adenosine (sAMS)**. **(A) **The flux ratio *f*_*MS *_of the mycobactin synthesis reaction as measured in the cell-fee reaction assay as a function of sAMS concentration [sAMS]. The calculated values (*solid line*) using the inhibition model given in Eq. 15 are in good agreement with the experimentally determined values (*squares*) [53]. **(B) **The calculated relative cell concentration *R*_*C *_of *Mycobacterium tuberculosis *as a function of sAMS inhibitor concentration [sAMS] in iron-deficient and iron-sufficient medium compared to the experimental data [53].

The inhibitory effect of sAMS on the mycobactin synthesis reaction has been experimentally studied in a cell-free *in vitro *reaction assay [53]. The measured inhibitory effect in the assay is quantified by the flux ratio of the mycobactin synthesis reaction as a function of sAMS concentration, which could be predicted by applying the developed inhibition model. We calculated the flux ratio *f*_*MS *_for a series of sAMS concentrations using the inhibition model given in Eq. 15, where we set the MbtA enzyme concentration ([*E*] in Eq. 15) to 20 nM. Figure 6A shows that there was an overall good agreement between the experimental and the simulated flux ratios, indicating that the inhibition model in Eq. 15 was capable of modeling the inhibitory effect of the target reaction.

We now turn to predicting the response of *M. tuberculosis *cells growing in iron-deficient GAST medium exposed to varying sAMS inhibitor concentrations. We used our framework and obtained the dose-response curve in Figure 6B (see Additional file 1: Section S7). The close agreement between predicted and experimental data [53] indicates that the mathematical framework was successful in coupling the three underlying models (inhibition, metabolic network, and population growth) to quantitatively predict the inhibitory effect of sAMS on *M. tuberculosis *growth in an iron-deficient medium. Moreover, to evaluate the agreement between the experimental data and their corresponding simulated values, we again performed a linear regression on the data [62]. The obtained slope (0.9223), intercept (0.0661), and coefficient of determination *R*^2 ^(0.9779) for the 22 data points in Figure 6B (for growth in iron-deficient medium) were commensurate with a *P *value of 4.9331 × 10^-18^, suggesting a strong and very similar relation between the simulated values and experimental data.

Similarly, we repeated the calculations for the inhibitory effect of sAMS in an iron-*sufficient *GAST medium. In this medium, siderophore sequestering is not an issue, because iron is freely available and the direct impact of inhibiting the mycobactin synthesis reaction should be negligible. Accordingly, we predicted that sAMS had no effect on *M. tuberculosis *growth in an iron-sufficient medium (see Additional file 1: Section S7). Figure 6B shows that our predictions matched the experimental data under relatively low sAMS concentration (<10 μM). At higher inhibitor concentration, however, it is speculated that the growth of *M. tuberculosis *in iron-sufficient medium is inhibited by sAMS through some other unknown mechanism [53]. Since this inhibitory mechanism is not accounted for in our model, we could not capture this feature. The modeling framework is thus quite powerful when the mechanism of inhibition is known. However, as illustrated, it cannot prospectively predict alternate binding of inhibitors or other cellular inhibition mechanisms not explicitly detailed in the model.

#### Sensitivity Analysis of Parameter Values

**Figure 7:**
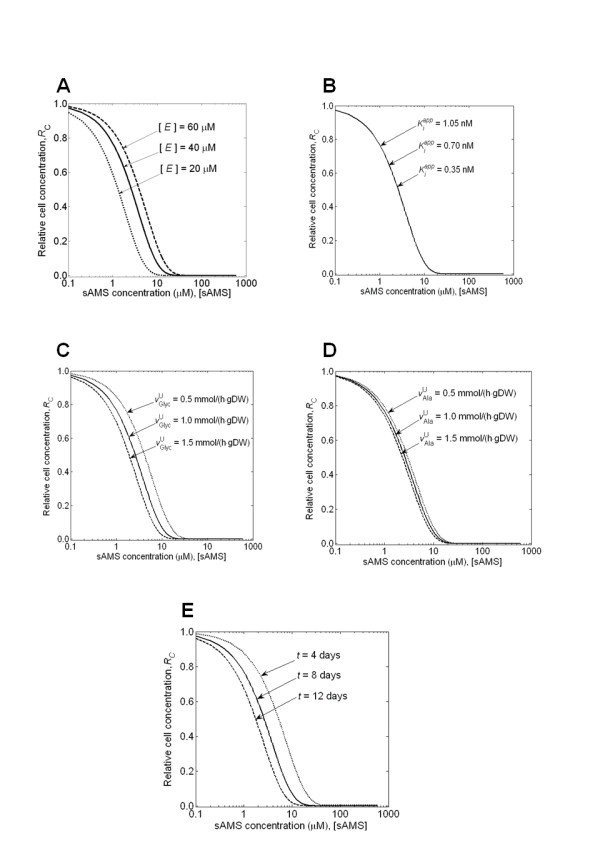
**The influence of the parameter values on the calculated dose-response curve**. Sensitivity analysis of the calculated relative cell concentration expressed as the ratio of inhibitor-present to inhibitor-free cell concentration of *Mycobacterium tuberculosis *after an 8-day growth period as a function of 5'-*O*-(*N*-salicylsulfamoyl) adenosine (sAMS) concentration. The analysis was performed for the **(A) **intracellular MbtA-enzyme concentration [*E*]; **(B) **apparent reaction rate constant ; **(C) **upper limit of glycerol uptake rate ; **(D) **upper limit of glycerol uptake rate ; and **(E) **time length of cellular growth *t*, which were each set to its original parameter value (*solid line*), the value increased by 50% (*dashed line*), and decreased by 50% (*dotted line*).

Table 3 summarizes the five parameters used to model growth inhibition by sAMS. Four of the five parameters were obtained from the literature, and the remaining parameter, intracellular MbtA enzyme concentration [*E*] in Eq. 15, was determined by matching experimental growth data. Figures 7A-E show the calculated dose-response curves when we increased and decreased each parameter by 50% in an iron-deficient medium. The curves were similar to each other and indicated that the final results were not critically dependent on our choice of parameter values. In Figure 7A, the dose-response curves associated with variations of the intracellular enzyme concentration [*E*] were consistent with the intuition that inhibiting higher concentrations of the enzyme requires additional inhibitor. The relatively high sensitivity of the calculated dose-response to the concentration of the MbtA enzyme in the cell [*E*] stems from this enzyme being the direct target of the sAMS inhibitor. Changes of [*E*] directly affect the required amount of sAMS to achieve a given level of inhibition, causing this parameter to strongly influence the dose-response curve. Figure 7B indicates that  had no effect on the calculated curves. Figure 7C shows that the upper limit of glycerol uptake () had a relatively large effect on the calculated curves. This is because glycerol is the major carbon source for *M. tuberculosis *and, therefore, changes of its uptake limit  directly affect the calculated growth rate of *M. tuberculosis *and the calculated dose-response curve. Figure 7D illustrates that the upper limit of alanine uptake () had a smaller effect than that of glycerol uptake () in Figure 7C, suggesting that, between the two carbon sources, alanine was not as important as glycerol for cellular growth. Table 4 shows the calculated sensitivity coefficients for all parameters, which also reinforced the observations that *1*) [*E*], , and t had noticeable effects on the calculated dose-response curves; 2)  had a relatively small effect; and 3)  had almost no effect. Among the parameters with large effects, time *t *could not be considered to be associated with any experimental variation. The sensitivity analysis identified the most critical parameters of the model to be [*E*] and , albeit at different inhibitor concentrations. We could further improve our framework by minimizing the number of matched parameter by directly obtaining values for [*E*] and  through experiments measuring intracellular enzyme activity [63] and nutrient uptake rate [65].

**Table 3:** Model parameters for cell growth inhibition by sAMS.

**Group**	**Parameter** **Annotation**	**Model**	**Eq.**	**Source of the value**
I	: apparent reaction rate constant	Inhibition Model	15	Set to be 0.7 nM from [53]
	
	: upper limit of glycerol uptake	Metabolic Network	-	Set to be 1 mmol/(h·gDW) from [42]
	
	: upper limit of alanine uptake	Metabolic Network	-	Set to be 1 mmol/(h·gDW) from [42]
	
	*t*: time length of cellular growth	Population Growth Model	17	Set to be 8 days from [53]

II	[*E*]: intracellular MbtA concentration	Inhibition Model	15	Obtained by matching experimental data

All parameter values were selected from the literature except for the intracellular MbtA enzyme concentration [*E*], which was obtained by matching experimental data.

**Table 4:** Sensitivity coefficients for the parameters in modeling sAMS inhibition.

**Parameter *p***	**Sensitivity Coefficient as a Function of [sAMS]**				
	
	**0.1 μM**	**1 μM**	**10 μM**	**100 μM**	**500 μM**
	0.0000	0.0000	0.0000	0.0000	0.0000

	-0.0197	-0.1966	-1.9662	-7.8648	-7.8648

	-0.0067	-0.0670	-0.6703	-2.6812	-2.6812

*t*	-0.0264	-0.2636	-2.6365	-10.5460	-10.5460

[*E*]	0.0264	0.2636	2.6365	0.0000	0.0000

The sensitivity coefficient  was calculated from a numerical estimate of , where *R*_*C *_is the relative cell concentration, defined as the ratio of inhibitor-present to inhibitor-free cell concentrations, and *p *represents the analyzed parameter.

## Discussion

We developed a mathematical framework connecting kinetic models of enzyme inhibition with metabolic network analysis and a population growth model. The three components correspond to the three major steps through which a metabolic inhibitor affects bacterial growth. First, the inhibition model describes how the particular inhibitor affects the enzyme kinetics and the flux(es) of one or more metabolic reactions. Second, the metabolic network analysis connects the changes in the affected metabolite flux(es) to the growth rate of the organism. Finally, the population growth model takes the altered growth rate and converts it to an effective bacterial cell concentration. This framework allowed us to quantitatively simulate the effect of two distinct metabolic inhibitors on *in vitro *bacterial growth under different nutritional conditions.

We applied this framework to model the effect of two separate metabolic inhibitors, 3-NP and sAMS, on the growth of *M. tuberculosis *cells on propionate medium and on iron-deficient GAST medium, respectively. Both reactions affected by these two inhibitors are required for the survival of the pathogen in the host environment and could potentially become important therapeutic targets. 3-NP inhibits key reactions in the glyoxylate shunt and the methylcitrate cycle, effectively blocking the utilization of fatty acids, the major carbon source of *M. tuberculosis *in the host environment [45,46]. sAMS inhibits the synthesis of mycobactin, which is required for iron uptake of *M. tuberculosis *within an iron-deficient host environment [53]. Our model was capable of quantitatively reproducing the experimentally determined dose-response curves for both inhibitors. Thus, with the proposed mathematical framework, we could analyze the studied system under conditions matching the experimental protocols as they relate to metabolism. We accounted for the underlying kinetics of the inhibition, how this was translated via the metabolic network analysis to metabolite flow and biomass accumulation, and to the growth of the cell population that was used as the experimental readout for drug inhibition. We noted, however, that certain cellular processes or responses that impact drug action in the cell, for example, adaptive responses in the form of altered gene expression of metabolic enzyme and activated drug efflux transport, were not accounted for in the proposed modeling scheme. These processes may play important roles and may need to be accounted for when modeling inhibitor effects of other than those of 3-NP and sAMS.

In this work, the mathematical framework was used to model an inhibitor's effect on cellular growth of a pathogen in different *in vitro *environments designed to duplicate aspects of the nutritional conditions encountered in the host. However, intracellular pathogens have complex interactions with their hosts [32] and the conclusions drawn from an *in vitro *environment may not be operative in the *in vivo *host environment [66]. The current models in our framework could be coupled to other models that, in turn, determine the medium content by simulating the metabolic nutrients available in a human macrophage cell. Such an embedding of the current modeling framework within other schemes could be used to add further biological complexity to the existing computational platform. Enzyme activity could be further coupled to a gene expression model to modulate protein/enzyme function according to microarray gene expression data [67]. The implementation of additional models is only limited by the availability of experimental data with which to perform rigorous parameter testing and prediction validation.

For the two inhibitors studied, the essentiality of the protein targets was a necessary condition. Essentiality of a gene can be imparted by the network itself or any other condition that alters of restricts the flux of metabolites in the network. Thus, some genes become essential only under specific nutritional conditions, while others may become essential when one or more nonessential genes are knocked out. It is also possible to envision certain scenarios where drugs affecting parts of the metabolic network induce essentiality to uninhibited enzymes in the network. Quantitative models, such as the one developed here, could be used to rapidly investigate such conditions and assist future experimental studies. For example, using our framework, we suggested that 3-NP was effective in fatty acid medium but not in glucose medium (data not shown), which was supported by experimental observations [46]. In addition to the two inhibitors examined in this study, our calculations also suggested that the inhibitor targeting protein TrpD (a drug target discussed in [42]) will only be effective when tryptophan is absent from the medium (data not shown). This observation calls for further experimental verification.

The current work introduces a systems biology approach using enzyme kinetics, metabolic networks, and population growth models that is capable of capturing the essential chemical and biological variability of the system under study. This enabled us to simulate and understand the underlying chemical and biological factors that give rise to the experimental observables, in this case growth inhibition of *M. tuberculosis *cells. Our results suggest that this type of inclusive modeling approach would be valuable in proposing new experimental studies by extending, combining, and exploring novel chemical and biological inhibition concepts.

## Conclusion

We implemented a systems biology framework, which combines detailed models of enzyme kinetics, a complete metabolic network analysis, and a cell population growth model, to represent and understand cellular growth inhibition in response to drugs. We used this mathematical framework to simulate two separate inhibition mechanisms for the growth of *M. tuberculosis *cells in an *in vitro *environment, which was modeled to represent the nutritional challenges encountered in a host cell. We calculated dose-response curves corresponding to the cellular growth versus drug concentration for the growth in a medium whose carbon source was restricted to fatty acids and was infused with varying concentrations of the 3-NP inhibitor. Similarly, we obtained dose-response curves for cells grown in medium with low-iron concentration and exposed to different amounts of the sAMS inhibitor. These results quantitatively reproduced experimentally measured dose-response curves, ranging over three orders of magnitude in inhibitor concentration. The ability of the proposed models to capture *in vitro *drug inhibition confirms that relevant features of intracellular metabolism of *M. tuberculosis *can be modeled by a metabolic network-based framework.

## Authors' contributions

All authors contributed to the design and coordination of the study. XF performed the computational implementations, and XF and AW prepared the original draft, which was revised by JR. All authors read and approved the final manuscript.

## Supplementary Material

Additional file 1 **Supplementary information**. Supplementary information provides the details for metabolic network modification and intermediate results during computation. Click here for file

Additional file 2 **Metabolic network used to model the inhibitory effect of 3-nitropropionate**. The network file is in format of Systems Biology Makeup Language. Click here for file

Additional file 3 **Metabolic network used to model the inhibitory effect of 5'-*O*-(*N*-salicylsulfamoyl) adenosine**. The network file is in format of Systems Biology Makeup Language. Click here for file
