# Supplementary material for: A systems biology framework for modeling metabolic enzyme inhibition of Mycobacterium tuberculosis
Source: BMC Syst Biol. 2009 Sep 15;3:92. doi: 10.1186/1752-0509-3-92 (PMC2759933; doi:10.1186/1752-0509-3-92)
Supplement: Additional file 1 — Supplementary information. Supplementary information provides the details for metabolic network modification and intermediate results during computation. [file 1752-0509-3-92-S1.pdf]

Supplementary Material

A Systems Biology Framework for Modelling Metabolic Enzyme Inhibition of *Mycobacterium tuberculosis*

Xin Fang, Anders Wallqvist, and Jaques Reifman*

Supplementary Information

S1. Modification of Metabolic Network

We made some modifications on the metabolic network developed by Jamshidi and Palsson [1] for *Mycobacterium tuberculosis*. Since the original set of reactions and metabolites lack the methylcitrate cycle [2, 3], we added the following reactions and their components to the network: methylcitrate synthase, methylcitrate dehydratase, and methylisocitrate lyase (MCL). In addition, because experimental data suggest that the methylmalonyl-coenzyme A (CoA) pathway may not function when *M. tuberculosis* grows in an odd-chain fatty acid medium [3], comparable to the medium studied here, and that the methylmalonyl-CoA pathway has a similar function to the methylcitrate cycle, we blocked this pathway by deleting the reaction catalyzed by propionyl-CoA carboxylase.

S2. Obtaining Undetermined Parameter Values in Modelling Cell Growth Inhibition by 3-NP

We obtained the values for $[C']$ $(t=0)$, V_m, and K_m' by reproducing the experimental cell concentrations of *M. tuberculosis* grown in an inhibitor-free propionate medium. We placed no constraints on the fluxes of the target reactions, isocitrate lyase (ICL) and MCL, and applied flux balance analysis (FBA) to the metabolic network of this organism. Figures S1A and S1B (solid line) show the resulting inhibitor-free biomass growth rate μ and propionate uptake rate $v_{C,}$ respectively, under different upper-limit constraints on propionate uptake v_C^U. We then used these results to solve the population growth model (Eqs. 10-13 in Main Text) and estimate the experimental of *M. tuberculosis* in an inhibitor-free propionate medium. We systematically

manipulated the values for these three parameters until the estimated cell concentrations reproduce the experimental ones.

To obtain the value of $K_{SUC,ICL1}$, we varied the its value until we obtained close agreement between experimental and predicted growth data by using our mathematical framework. When we set the value to 1.5 mM, we calculated the corresponding biomass growth rates μ and propionate uptake rates v_C, respectively, as obtained from the FBA of the metabolic network, for growth with 0.025 mM 3-NP inhibitor (dashed line in Figure S1A and S1B). We then applied the obtained biomass growth rates μ and propionate uptake rates v_C into the population growth model (Eqs. 10-13 in Main Text), and predicted growth data which agreed with experimental data.

S3. Verification of Essentiality of Target Reactions in Modelling Cell Growth Inhibition by 3-NP

To verify that the two 3-NP-inhibited reactions, ICL and MCL, are necessary for the growth of *M. tuberculosis* in propionate medium, we set the fluxes associated with these reactions to zero and applying the FBA. The dotted line in Figure S1A and S1B show that the resultant biomass growth rate μ and propionate uptake rate v_C, respectively, for the growth of mutant *Δicl1Δicl2* were zero. Accordingly, the function g used in the population growth model (Eq. 12 in Main Text) was identical to zero, and this model predicted complete lack of bacterial growth. We did not consider maintenance fluxes (i.e., fluxes used for the viability of the bacterium but not used for growth), because FBA assumes that all possible metabolic fluxes are used to maximize cellular growth.

S4. Growth Predictions in Modelling Cell Growth Inhibition by 3-NP

We used the mathematical framework to predict the growth of *M. tuberculosis* at three 3-NP inhibitor concentrations (0.050, 0.100, and 0.200 mM). We used the flux ratios shown in Figure S2A obtained from Eqs. 8 and 9 in Main Text to constrain the fluxes of the ICL and MCL reactions in the metabolic network. Next, we applied the FBA to obtain μ and v_{C} , for each of the three inhibitor concentrations, as shown in Figure S2B and S2C, respectively. Finally, we applied these rates for the population growth model (Eqs. 10-13 in Main Text) to simulate *M. tuberculosis* growth at the three different 3-NP concentrations.

S5. Obtaining Undetermined Parameter Values in Modelling Cell Growth Inhibition by sAMS

When we set [*E*] = 40 μM, we first used Eq. 14 in Main Text to calculate the flux ratio f_{MS} for this particular value of [*E*] at the given [sAMS] of 1.7 μM. Then, with the obtained f_{MS}, we applied the FBA to the metabolic network specific to iron-deficient GAST medium to calculate the inhibitor-free biomass growth rate μ^0 and the inhibitor-present rate μ. Given these growth rates, next, we used Eq. 16 in Main Text and calculated the relative cell concentration R_C of 0.47 which was close to the selected value 0.49.

S6. Verification of Essentiality of Target Reactions in Modelling Cell Growth Inhibition by sAMS

To verify the essentiality of the targeted reaction, mycobactin synthesis in the presence of sAMS, for cellular growth of *M. tuberculosis* in iron-deficient GAST medium, we set the flux of the mycobactin synthesis reaction to zero and applied the FBA to the metabolic network, which, as

expected, yielded a biomass growth rate μ of zero. We applied this growth rate for the population growth model (Eq. 16 in Main Text) and obtained a relative cell concentration R_C of zero.

S7. Growth Predictions in Modelling Cell Growth Inhibition by sAMS

To predict the response of *M. tuberculosis* cells growing in iron-deficient GAST medium exposed to varying sAMS inhibitor concentrations, we first used the inhibition model (Eq. 14 in Main Text), with the intracellular MbtA-enzyme concentration set to 40 μM, to calculate the flux ratio f_{MS} of the mycobactin synthesis reaction at varying sAMS concentrations (Figure S3A). Next, we applied the FBA method to estimate the biomass growth rate μ as a function of f_{MS} (Figure S3B). Finally, we used the population growth model (Eq. 16 in Main Text) to obtain the relative cell concentration R_C as a function of biomass growth rate μ (Figure S3C). Using the functional dependencies given in Figures S3A-S3*C*, we mapped the relationship between inhibitor concentration [sAMS] and relative cell concentration R_C of *M. tuberculosis*, yielding the dose-response curve.

To calculate the effect of sAMS on *M. tuberculosis* growth in an iron-*sufficient* GAST medium, we applied the FBA of the metabolic network specific to this medium to estimate the biomass growth rate μ as a function of f_{MS}, and the resulting biomass growth rates were quite different from those of the iron-deficient medium. Figure S3B shows that the resultant biomass growth rate μ in iron-sufficient medium was always equal to the inhibitor-free growth rate μ^0. Accordingly, the relative cell concentration R_C from the population growth model (Eq. 16 in Main Text) was also always equal to one. Thus, we predicted that sAMS had no effect on *M. tuberculosis* growth in an iron-sufficient medium.

References

1. Jamshidi N, Palsson BO: **Investigating the metabolic capabilities of Mycobacterium tuberculosis H37Rv using the in silico strain iNJ661 and proposing alternative drug targets.** *BMC Syst Biol* 2007, **1:**26.
2. Gould TA, van de Langemheen H, Munoz-Elias EJ, McKinney JD, Sacchettini JC: **Dual role of isocitrate lyase 1 in the glyoxylate and methylcitrate cycles in Mycobacterium tuberculosis.** *Mol Microbiol* 2006, **61:**940-947.
3. Munoz-Elias EJ, Upton AM, Cherian J, McKinney JD: **Role of the methylcitrate cycle in Mycobacterium tuberculosis metabolism, intracellular growth, and virulence.** *Mol Microbiol* 2006, **60:**1109-1122.

Figures

Figure S1 - Intermediate results to obtain parameters and verify reaction essentiality in the study of 3-nitropropionate (3-NP).

Intermediate results were from the metabolic network analysis used to obtain parameter values and verify essentiality of target reactions in the study of the inhibitory effects of 3-nitropropionate (3-NP): **(A)** Biomass growth rate μ versus the upper limit of propionate uptake v_C^U for the growth of *Mycobacterium tuberculosis* in an inhibitor-free medium (*solid line*), in medium infused with 0.025 mM 3-NP (*dashed line*), and for the growth of the *Δicl1 Δicl2* mutant bacterium (*dotted line*); **(B)** Propionate uptake rate v_C as a function of the upper limit of propionate uptake v_C^U for the growth of *M. tuberculosis* in inhibitor-free medium (*solid line*), in medium infused with 0.025 mM 3-NP (*dashed line*), and for the growth of the *Δicl1 Δicl2* mutant bacterium (*dotted line*).

Figure S2 - Intermediate results to predict growth in the study of 3-nitropropionate (3-NP).

Intermediate results were from the inhibition model (**A**) and metabolic network analysis (**B**, **C**) used to predict the growth of *Mycobacterium tuberculosis* in the presence of varying amounts of 3-nitropropionate (3-NP): **(A)** Flux ratios *f* of the isocitrate lyase (ICL) reaction (*solid line*) and methylisocitrate lyase (MCL) reaction (*dashed line*) as a function of different 3-NP concentrations [3-NP]; **(B)** Biomass growth rate μ versus the upper limit of propionate uptake v_C^U for *M. tuberculosis* in medium containing 0.05 mM (*solid line*), 0.1 mM (*dashed line*), and 0.2

mM (*dotted line*) 3-NP; **(C)** Propionate uptake rate v_C as a function of the upper limit of propionate uptake v_C^U of *M. tuberculosis* for the same conditions as in (*B*).

Figure S3: Intermediate results in the study of 5'-*O*-(*N*-salicylsulfamoyl)adenosine (sAMS).

Intermediate results were obtained from the inhibition model (**A**), metabolic network analysis (**B**), and population growth model (**C**) used to study the inhibitory effects of 5'-*O*-(*N*-salicylsulfamoyl)adenosine (sAMS) on *Mycobacterium tuberculosis* growth. **(A)** Flux ratio f_{MS} of the mycobactin synthesis reaction, i.e., the target reaction that is inhibited by sAMS, as a function of the concentration of sAMS inhibitor; **(B)** Biomass growth rate μ versus flux ratio f_{MS} in iron-deficient and iron-sufficient medium; and **(C)** Relative cell concentration R_C as a function of biomass growth rate μ. The maximum growth rate given on the *x*-axis corresponds to the inhibitor-free biomass growth rate μ^0 shown in (**B**).

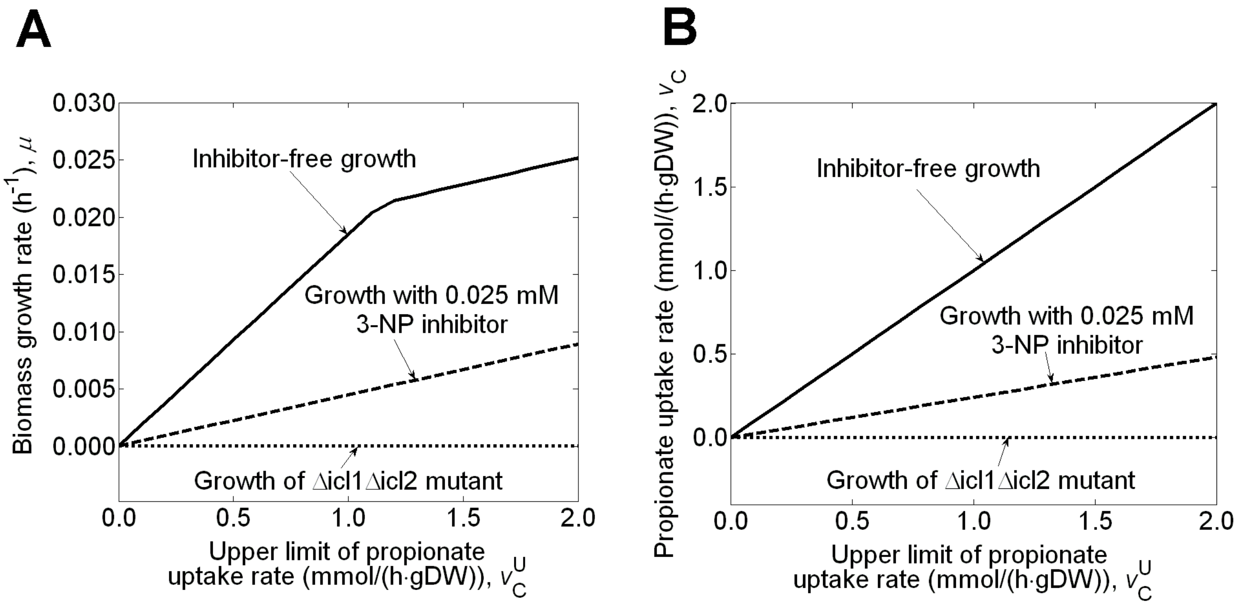
A
Biomass growth rate (h^{-1}), μ
0.030
0.025
0.020
0.015
0.010
0.005
0.000
Inhibitor-free growth
Growth with 0.025 mM 3-NP inhibitor
Growth of ∆icl1∆icl2 mutant
0.0
0.5
1.0
1.5
2.0
Upper limit of propionate uptake rate (mmol/(h·gDW)), v_C^U
B
Propionate uptake rate (mmol/(h·gDW)), v_C
2.0
1.5
1.0
0.5
0.0
Inhibitor-free growth
Growth with 0.025 mM 3-NP inhibitor
Growth of ∆icl1∆icl2 mutant
0.0
0.5
1.0
1.5
2.0
Upper limit of propionate uptake rate (mmol/(h·gDW)), v_C^U

Figure S1

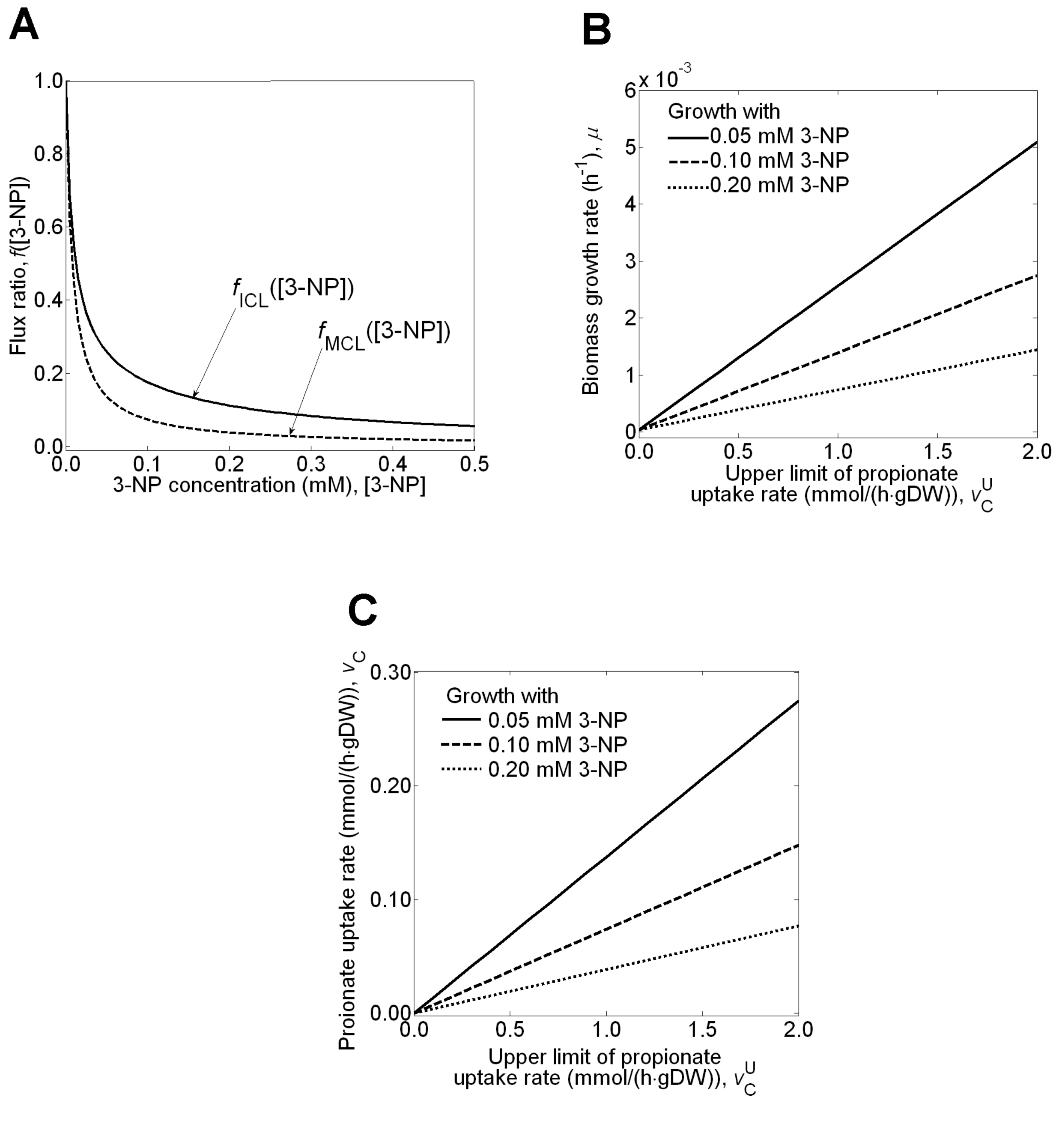
A
1.0
0.8
0.6
0.4
0.2
0.0
Flux ratio, f([3-NP])
f_ICL([3-NP])
f_MCL([3-NP])
0.0
0.1
0.2
0.3
0.4
0.5
3-NP concentration (mM), [3-NP]
B
x 10^-3
6
5
4
3
2
1
0
Biomass growth rate (h^-1), μ
Growth with
0.05 mM 3-NP
0.10 mM 3-NP
0.20 mM 3-NP
0.0
0.5
1.0
1.5
2.0
Upper limit of propionate
uptake rate (mmol/(h·gDW)), v_C^U
C
0.30
0.20
0.10
0.00
Proionate uptake rate (mmol/(h·gDW)), v_C
Growth with
0.05 mM 3-NP
0.10 mM 3-NP
0.20 mM 3-NP
0.0
0.5
1.0
1.5
2.0
Upper limit of propionate
uptake rate (mmol/(h·gDW)), v_C^U

Figure S2

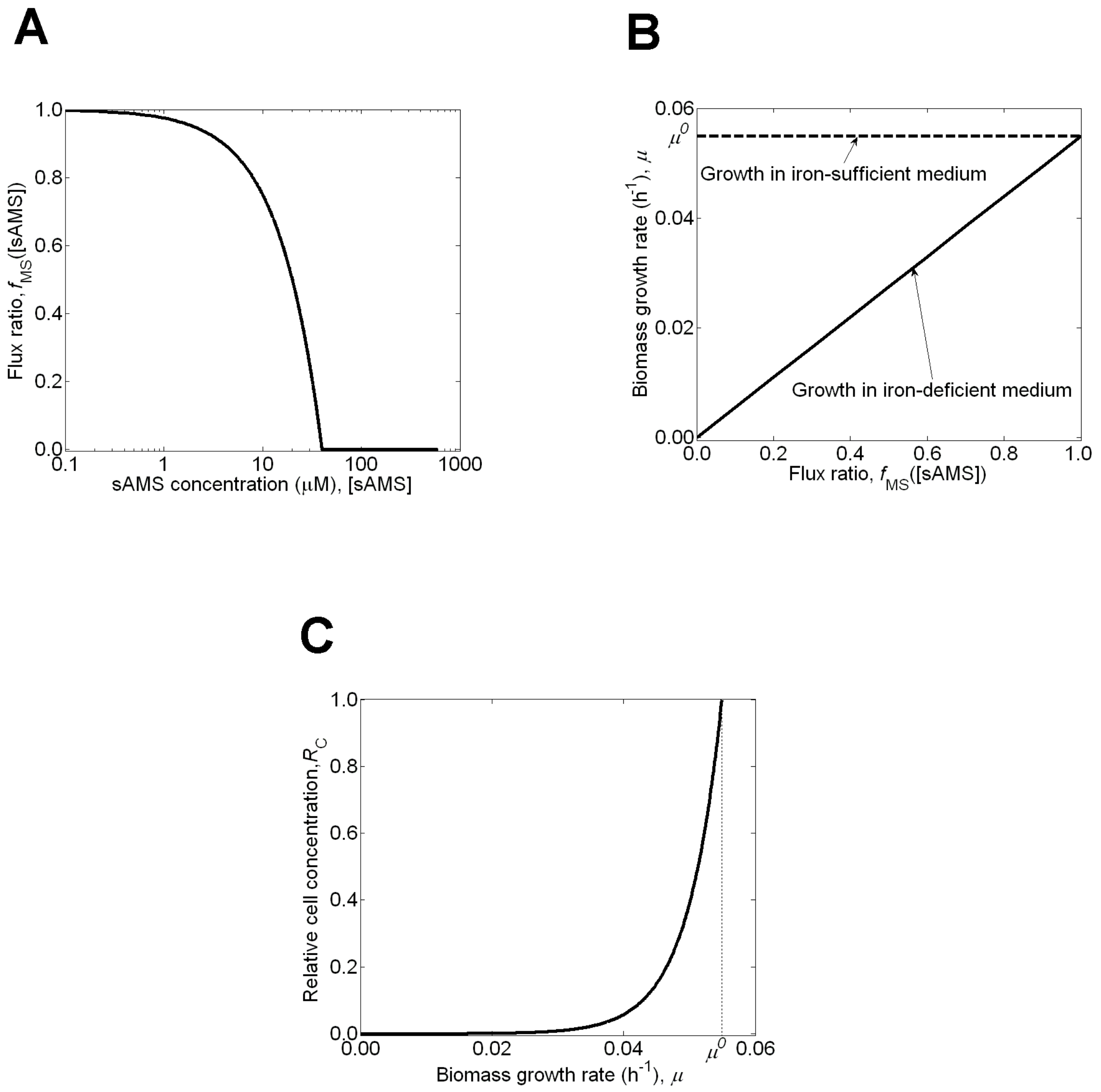
A
Flux ratio, f_MS([sAMS])
sAMS concentration (μM), [sAMS]
B
Biomass growth rate (h^-1), μ
μ^0
Growth in iron-sufficient medium
Growth in iron-deficient medium
Flux ratio, f_MS([sAMS])
C
Relative cell concentration, R_C
Biomass growth rate (h^-1), μ

Figure S3
